# Effects of Perivitelline Fluid Obtained from Horseshoe Crab on The Proliferation and Genotoxicity of Dental Pulp Stem Cells

**DOI:** 10.22074/cellj.2016.3726

**Published:** 2015-07-11

**Authors:** Marahaini Musa, Khadijah Mohd Ali, Thirumulu Ponnuraj Kannan, Ahmad Azlina, Nor Shamsuria Omar, Anil Chatterji, Khairani Idah Mokhtar

**Affiliations:** 1 School of Dental Sciences, Universiti Sains Malaysia, Kelantan, Malaysia; 2 Human Genome Centre, School of Medical Sciences, Universiti Sains Malaysia, Kelantan, Malaysia; 3 Institute of Tropical Aquaculture (AQUATROP), University Malaysia Terengganu, Terengganu, Malaysia; 4 National Institute of Oceanography (NIO), Dona Paula, India; 5 Kulliyah of Dentistry, International Islamic University of Malaysia, Jalan Sultan Ahmad Shah, Pahang, Malaysia

**Keywords:** Horseshoe Crabs, Proliferation, Genotoxicity, Mutagenicity

## Abstract

**Objective:**

Perivitelline fluid (PVF) of the horseshoe crab embryo has been reported to possess an important role during embryogenesis by promoting cell proliferation. This study aims to evaluate the effect of PVF on the proliferation, chromosome aberration (CA) and mutagenicity of the dental pulp stem cells (DPSCs).

**Materials and Methods:**

This is an *in vitro* experimental study. PVF samples were collected from horseshoe crabs from beaches in Malaysia and the crude extract was prepared. DPSCs were treated with different concentrations of PVF crude extract in an 3-(4,5-dimethylthiazol-2-yl)-2,5-diphenyl tetrazolium bromide (MTT) assay (cytotoxicity test). We choose two inhibitory concentrations (IC_50_ and IC_25_) and two PVF concentrations which produced more cell viability compared to a negative control (100%) for further tests. Quantitative analysis of the proliferation activity of PVF was studied using the AlamarBlue®assay for 10 days. Population doubling times (PDTs) of the treatment groups were calculated from this assay. Genotoxicity was evaluated based on the CA and Ames tests. Statistical analysis was carried out using independent t test to calculate significant differences in the PDT and mitotic indices in the CA test between the treatment and negative control groups. Significant differences in the data were P<0.05.

**Results:**

A total of four PVF concentrations retrieved from the MTT assay were 26.887 mg/ml (IC_50_), 14.093 mg/ml (IC_25_), 0.278 mg/ml (102% cell viability) and 0.019 mg/ml (102.5% cell viability). According to the AlamarBlue®assay, these PVF groups produced comparable proliferation activities compared to the negative (untreated) control. PDTs between PVF groups and the negative control were insignificantly different (P>0.05). No significant aberrations in chromosomes were observed in the PVF groups and the Ames test on the PVF showed the absence of significant positive results.

**Conclusion:**

PVF from horseshoe crabs produced insignificant proliferative activity on treated DPSCs. The PVF was non-genotoxic based on the CA and Ames tests.

## Introduction

Horseshoe crabs which are the closest living relatives of the trilobites have survived for more than 200 million years (1, 2). In the present day, the population of horseshoe crabs can be found in only two regions of the world. Three species of horseshoe crabs, namely, *Tachypleus tridentatus* (*T. tridentatus*), *Tachypleus gigas (T. gigas)* and *Carcinoscorpius rotundicauda*, occupy Asian coastal waters from India to Japan, south to Malaysia and Indonesia, including waters around the Dutch East Indies and the Philippine Islands. Another species of horseshoe crab (*Limulus polyphemus*) is found along the Atlantic coastline of North America from Maine to the Yucatan, from about 19˚N to 42˚N (1). The Asian horseshoe crab *T. tridentatus* and the coastal horseshoe crab *T. gigas* populate sandy to muddy habitats (3-5).

Besides the hemolymph, other products of horseshoe crabs such as perivitelline fluid (PVF) have been shown to possess essential medicinal properties. PVF refers to fluid that fills the perivitelline space (a space between the newly formed inner egg membrane and embryo) during early developmental stages of the horseshoe crab embryo (6). PVF contains proteins such as hemagglutinins and hemocyanins which may play an important role during embryogenesis (7, 8).

Among the identified adult stem cells are postnatal stem cells in human dental pulp, called dental pulp stem cells (DPSCs) (9). DPSCs refer to a multipotent mesenchymal type of stem cell that has the potential to differentiate into various types of other cells including cardiomyocytes for repair of damaged cardiac tissue following a heart attack (10), neurons to generate nerve and brain tissue (11), myocytes for muscle repair (12) and osteocytes for bone generation (13). The multipotency of this stem cell serves as an indication that this tissue has tremendous potential for clinical applications. Thus it offers researchers the opportunity to elucidate the mechanisms at cellular and molecular levels that operate during development and regeneration of dental and other craniofacial structures (14). Stem cell research has gained more attention over the years due to its special characteristics, namely, the ability to proliferate and differentiate into different types of cells. Previous studies have shown the unique ability of PVF to induce cell proliferation, thus potentially serving as a valuable supplement to stem cells.

The two main concerns in the safety assessment of drugs and chemicals are the mutagenic and genotoxic potential of particular agents (15, 16). Genetic toxicity assessment is performed to determine the ability of certain agents to induce any of three general types of changes (mutations) in genetic material (DNA) namely genes, chromosomes and genomes. Genotoxicity can lead to significant, irreversible effects upon human health and genotoxic damage has been proven as a crucial factor for carcinogenesis. The significant effect of genotoxicity can be seen in the onset of birth abnormalities and fetal death. The three types of mutations mentioned previously may involve either of the two types of tissues. They are germ cells (sperm or eggs) and somatic cells. Genotoxicity test results are often taken as indicators for the mutagenic effectsof chemicals (17).

In this study, we studied the effects of PVF obtained from Malaysian horseshoe crabs on DPSCs to evaluate their proliferation activity. Prior to the proliferation assay (AlamarBlue® assay), the cytotoxicity assessment of PVF from horseshoe crabs using the 3-[4,5-dimethylthiazol-2-yl]-2,5- diphenyl tetrazolium bromide (MTT) assay was conducted. Genotoxicity was evaluated based on the chromosome aberration (CA) and Ames tests.

## Materials and Methods

### Study design

This was an *in vitro* experimental study carried out on DPSCs.

### Cell culture

DPSCs from AllCells (USA, cat no. DP004F) were cultured in mesenchymal stem cell (MSC) basal medium (AllCells, cat no. MSC-002) supplemented with MSC stimulatory supplement (AllCells, cat no. MSC-003) and incubated at 37˚C in a 5% CO_2_ humidified incubator until confluent.

### Perivitelline fluid

Fertilized eggs from the horseshoe crab were collected from the nests on a sandy beach in Kuantan, Malaysia. The eggs were processed at Aquatrop Laboratory at the University Malaysia Terengganu (UMT), Malaysia. Eggs were incubated at a constant temperature of 29 ± 1˚C in artificial incubators until they became transparent and showed the movement of trilobite larvae (18). Further processing of the fertilized eggs and purification steps were performed according to Chatterji et al. (18). The freeze-dried PVF was stored at -70˚C until use. For preparation of the PVF extract, the test sample was mixed with 1 ml of phosphate-buffered saline (PBS, Invitrogen, UK) and further diluted to various concentrations using culture medium. The PVF extract was sterilized through a 0.25 μm syringe filter (Sartorius, UK). The extract was prepared fresh for each experiment.

### 3-[4,5-dimethylthiazol-2-yl]-2,5-diphenyl tetrazolium bromide (MTT) assay (cytotoxicity test)

The MTT assay was conducted according to Mosmann (19). Confluent DPSCs were washed with PBS) and trypsinized using 0.25% trypsin (Sigma-Aldrich, USA) solution. The culture medium then was added to the cells after which they were centrifuged at 1200 rpm for 5 minutes until a pellet was formed. The cells were counted and 1×10^3^ DPSCs were seeded into a 96-well plate. After overnight incubation, the PVF at different concentrations (45, 22.5, 11.25, 5.625, 2.813, 1.406, 0.703, 0.352 mg/ml) were added to the cells and incubated for 72 hours. Then, 10 μl of a 5 mg/ml MTT solution was pipetted into each well followed by a 4 hours incubation period after which 100 μl dimethyl sulfoxide (DMSO, Merck, USA) was added to each well to dissolve the insoluble formazan salt which formed as a result of mitochondrial activity of the viable cells. The absorbance in the plate was read using an enzyme-linked immunosorbent assay (ELISA) plate reader at 570 nm (Tecan, Switzerland). Each experiment was performed in triplicate. The percentages of relative cell viability with regards to control wells that contained cell culture medium without extracts (100%) were determined using the following formula:

Cell viability=[A]test/[A]control×100%

where [A]test is the absorbance of the test sample and [A]control is the absorbance of the control sample. A dose-inhibition graph was constructed using the data from the MTT assay. The inhibitory concentrations (IC_50_ and IC_25_) values were derived from the graph using ED50V10 software. Another two concentrations of PVF which produced higher cell viability compared to the control (100%) were chosen for consecutive tests.

AlamarBlue® assay Prior to treatment with the test material, we constructed a standard curve of DPSCs where different count of cells (15000, 7500, 3750, 1875, 937, 469, 234 and 117) were seeded in a 96-well plate and incubated overnight before 10 μl of 0.4% AlamarBlue® solution was added. Absorbance of each well was read at 570 and 600 nm using an ELISA plate reader. For the test, 1×10^3^ cells were seeded in a 96-well plate and incubated overnight before 100 μl of PVF extract of IC_50_, IC_25_ values and two concentrations that had higher cell viability compared to the control in the MTT assay were treated with cells. Addition of AlamarBlue® solution and absorbance reading were performed as mentioned once every two days for ten days. The experiment was conducted in triplicate. The percentage of reduction of each group was determined using the following formula:

$$\mathrm{Percentage reduction}=[(117.216)A_{570}-(80.586)A_{600}]/[(155.677){\mathrm{A΄}}_{600}-(14.652){\mathrm{A΄}}_{570}]\times 100\%$$

where 117.216; molar extinction coefficient of AlamarBlue® in the oxidized form at 600 nm, 80.586; molar extinction coefficient of AlamarBlue® in the oxidized form at 570 nm, 14.652; molar extinction coefficient of AlamarBlue® in the reduced form at 600 nm, 155.677; molar extinction coefficient of AlamarBlue® in the reduced form at 570 nm, A_600_; absorbance of test wells at 600 nm, A_570_: absorbance of test wells at 570 nm, A΄_600_; absorbance of negative control wells at 600 nm and A΄_570_; absorbance of negative control wells at 570 nm.

The graph of percentages of the reduction was constructed and we calculated the population doubling time (PDT) as the time taken for the cell to double of the treated DPSCs. The doubling time was calculated from times for doubling cell number in the log phase of the resultant growth curve (20).

### Chromosome aberration test

For the CA test of PVF on DPSCs, the protocol was followed according to the Organization for Economic Cooperation and Development (OECD) Test Guideline 473 (21). A total of 1×10^5^ DPSC cells were seeded in a 60 mm culture dish and incubated overnight before treatment with PVF (IC_50_, IC_25_ values and two concentrations with higher cell viability compared to the control in the MTT assay), concurrently with negative and positive controls for either 4 or 24 hours. Depending on the treatment condition, different positive controls were applied-mitomycin C (MMC) and cyclophosphamide monohydrate (CP, Merck, Germany) for treatment without and with addition of S9 mix (metabolic activation system), respectively.

For the 4-hour treatment, cells were treated with PVF extracts (concentrations which produced IC_50_, IC_25_ and two other concentrations which produced higher cell viability as compared to the control in the MTT assay), negative control (culture medium), and positive control (0.1 μg/ml MMC without S9 or 10 μg/ml CP with S9).

Then, the cells were washed with PBS and the culture medium was added. The cells were further incubated for 22 hours before the addition of a 1 μg/ml metaphase-arresting agent, Colcemid (Invitrogen, UK) for 2 hours. For 24 hours of treatment, the addition of PVF, negative control and positive control (0.05 μg/ml MMC) were done after overnight incubation following the seeding and colcemid was added to the cells 2 hours before the end of the 24-hour treatment duration. Following incubation, the cells were washed and subjected to hypotonic treatment using pre-warmed 0.075 M potassium chloride (Invitrogen, UK) for 50 minutes. Then, the cells were fixed using cold 3:1 methanol and acetic acid solution for 3 times followed by the preparation and staining of the slides using Giemsa stain (Sigma-Aldrich, USA) for 20 minutes. The metaphase spread was studied under ×100 magnification using a Nikon Eclipse E600 microscope (Nikon, Japan). A total of 100 metaphase chromosomes were analyzed per sample. Numerical and structural aberration was observed. The mitotic index (MI) of each treatment group which defines the ratio of cells in metaphase divided by the total number of cells observed in a population of cells was calculated using the following formula:

MI=(total number of metaphase/total number of cells)x100%

where total number of cells=1000

MI also acts as an indication of the degree of proliferation of that population (21). The CA test was carried out in duplicate.

### Ames salmonella/microsome mutagenicity assay (Ames test)

#### Bacterial strains

Two strains of *Salmonella typhimurium (S. typhimurium)* used in the study, TA98 and TA100, were obtained from Dr. T. Nohmi from the National Institute of Health Science, Tokyo, Japan. The strains were kept in 0.5 ml of 30% glycerol (Sigma-Aldrich, USA) and 0.5 ml of broth culture (Oxoid, UK) at -80˚C in an ultra-deep freezer (Sanyo, Japan) prior to use. Two selected strains of *S. typhimurium* (TA98 and TA100) were chosen to identify two types of mutations (base-pair substitution and frame shift).

#### Medium

The medium used was glucose minimal agar medium (GM agar) which consisted of 0.5% of Vogel-Bonner minimal medium E (V/B salts), 2% glucose (R < M Marketing, UK) and 1.5% agar (HiMedia, India) along with an overlay, top agar which comprised 0.6% agar and 0.6% NaCl (Sigma-Aldrich, USA) which contained a trace amount of 0.05 mM histidine (Merck, Germany) and 0.05 mM biotin (Merck, Germany) that allowed for a few cell divisions were used. The Vogel- Bonner minimal medium E comprised warm distilled water (about 50˚C), magnesium sulfate (Merck, Germany), citric acid monohydrate (Mallinckrodt, Mexico), potassium phosphate (dibasic) anhydrous (Ajax Finechem, Australia) and sodium ammonium phosphate (Merck, Germany). The nutrient broth (Oxoid, UK) was prepared along with nutrient agar.

#### Ames testing

A total of five different concentrations of PVF were prepared for the Ames test. The IC_25_ (inhibitory concentration from the MTT assay) with a concentration of 14.09 mg/ml PVF was used as the highest concentration. This concentration was additionally diluted using sterile double distilled water (ddH_2_O) to produce PVF concentrations of 7.045, 3.5225, 1.76125, 0.880625 mg/ml. The assays were performed according the OECD Test Guideline 471 (22). Two treatment conditions were applied in the test-with and without addition of metabolic activation system (S9 mix). Both positive and negative controls were tested concurrently in the assay. In the present study, ddH_2_O was used as the negative control while the selected positive controls were 4-nitro-o-phenylenediamine (4-NoPD, Acros Organics, USA) and sodium azide (NAN3, Acros Organics, USA) for strains TA98 and TA100, respectively.

For the test, 0.05 ml of PVF extracts, positive control or negative control (distilled water), 0.05 ml of Salmonella strain and either 0.5 ml of 0.1 M sodium phosphate (Mallinckrodt, Mexico) buffer (pH=7.4) or the S9 mix were added to the 13×100 mm sterile glass tube. The mixture then was incubated for 20 minutes at 37˚C. Then, 2 ml of molten top agar was added, gently mixed and quickly poured onto the surface of the GM agar plate. To ensure even distribution of the overlay agar, this step was conducted by swirling the plates quickly after the addition of the top agar onto the surface of the GM agar plates (23). After the solidification of the top agar, the plate was incubated in inverted position at 37˚C for 48 hours. The experiments were performed in triplicate. The analysis was performed by counting the number of the revertant colonies using a Colony Analyzer (Acolyte, UK) which was then compared to the number of spontaneous revertant colonies per plate.

### Statistical analysis

The statistical analysis of the differences in the PDTs and MI of the treatment groups as compared to the negative control were studied using the independent t test (two-tailed, assuming unequal variances, Microsoft Office Excel 2007, Microsoft Corp., Seattle, WA, USA). Significant differences in the MI data were represented by a p-value of less than 0.05.

Due to the insensitivity of two-fold rule for Salmonella strains with relatively high reversion frequencies (TA100, TA97, and TA102) and oversensitivity for chemicals with low reversion frequencies (TA1535 and TA1537), the statistical approach has been considered as unsuitable method to interpret the result of this test. Therefore, we used a non-statistical approach for analysis. Mortelmans and Zeiger (24) implemented the following criteria for the interpretation of Ames test results where by: i. positive: a compound is considered a mutagen if it produces a reproducible, dose-related increase in the number of revertant colonies in one or more strains, ii. negative: a compound is considered a non-mutagen if no dose-related increase in the number of revertant colonies is observed in at least two independent experiments and iii. inconclusive: if a compound cannot be identified clearly as a mutagen or a non-mutagen, the results are classified as inconclusive.

## Results

### Cytotoxicity test (MTT assay)

**Fig.1:**
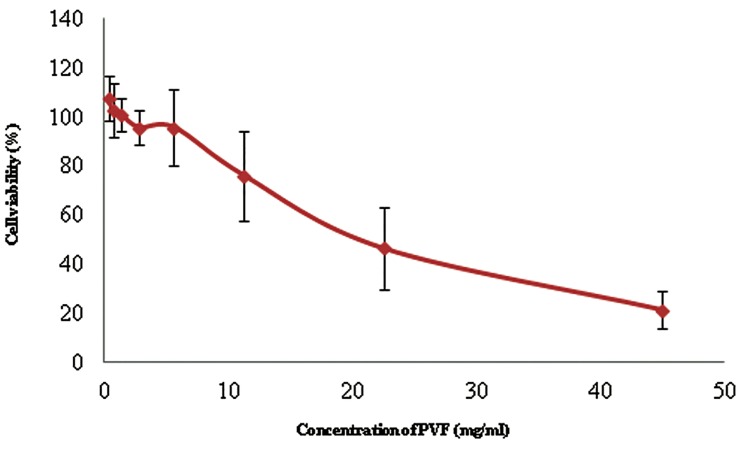
MTT assay results of perivitelline fluid (PVF). Higher concentration of PVF extracts produced lower cell viability. Reduction in the PVF concentrations produced higher percentages of viable cells. The error bars indicate SD values. MTT; 3-[4,5-dimethylthiazol-2-yl]-2,5-diphenyl tetrazolium bromide and SD; Standard deviation.

The cytotoxic effect of PVF was inversely proportional to the viability of tested DPSCs where the higher concentration of the extract produced lower cell viability compared to the diluted, lower concentration of PVF (Fig.1). As deduced from the graph, the IC_25_ and IC_50_ values were 14.093 and 26.887 mg/ml, respectively. The selected concentrations of PVF which produced more cell viability as compared to the control were 0.278 and 0.019 mg/ml where these concentrations resulted in 102 and 102.5% of viability of treated DPSCs.

There was no obvious increase in the number of viable DPSCs after treatment with PVF (Fig.1). Cells incubated with a much lower concentration of PVF were only able to produce a slight increase in the number of viable cells (less than 103%) compared to the untreated group. This might suggest the potential of PVF as an agent to promote cell proliferation even though the effect was quite minimal.

### AlamarBlue® assay

**Fig.2:**
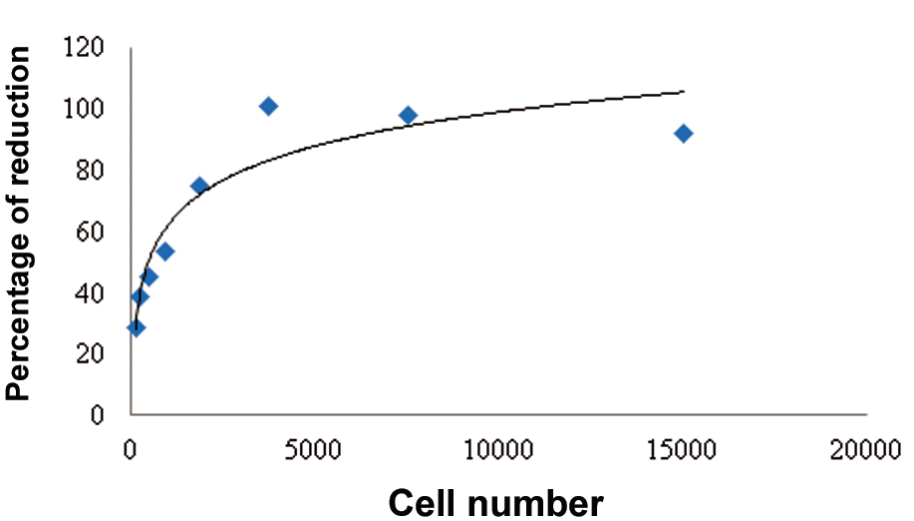
Standard curve of dental pulp stem cells (DPSCs).

**Fig.3:**
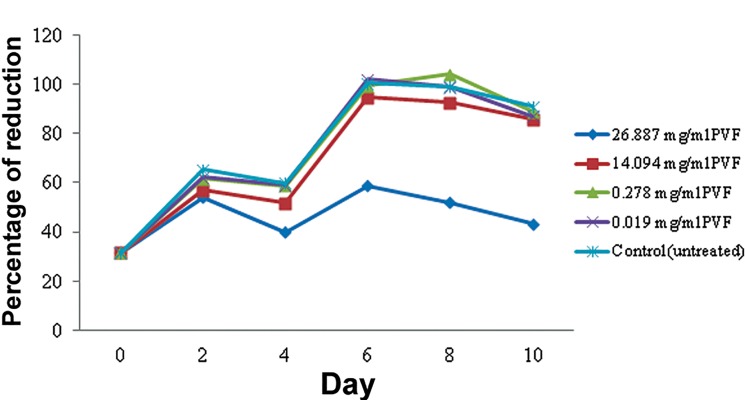
Proliferation effect of perivitelline fluid (PVF) on dental pulp stem cells (DPSCs) using the AlamarBlue® assay.

The standard curve of DPSCs is shown in figure 2 where higher cell number produced a greater percentage of AlamarBlue® dye reduction. The proliferation effects of PVF along with positive and negative controls on DPSCs for a 10-day period are shown in figure 3. All groups except for cells treated with 26.887 mg/ml PVF (IC_50_ value) produced an elevation in the percentages of reduction over time.

The IC_50_ concentration of PVF led to an almost constant reduction of AlamarBlue® dye throughout 10 days of the experiment. Even though the IC_50_ concentration seemed to not be toxic to cells because no major reduction of percentages was seen, that particular concentration of PVF did not support cell proliferation as opposed to other lower concentrations. For the IC_25_ concentration, the percentage of reduction was lower than other groups starting from days 0 to 8; however, the percentage became comparable at the last day of the experiment (day 10). As for other PVF groups, the cells treated with 0.278 and 0.019 mg/ml showed a comparable percentage of reduction to the negative control. However, 0.019 and 0.278 mg/ml PVF produced higher AlamarBlue® reduction than the untreated group on days6 and 8. On the last day of the test (day 10), there were similar percentages of all groups at the same level, except for 26.887 mg/ml of PVF.

Cells treated with 14.093, 0.278 and 0.019 mg/ml PVF produced comparable PDT with the negative control whereas a slight difference in PDT of cells treated with 26.887 mg/ml PVF was observed when compared to the negative control (Table 1). However, the results of the independent t test revealed no significant differences in PDTs of all treatment groups when compared to the negative control (P>0.05).

**Table 1:** Population doubling time (PDT) of cells treated with perivitelline fluid (PVF)


Description	PDT (SD) hours

PVF (26.887 mg/ml)	28.45(10.60)
PVF (14.093 mg/ml)	12.39(1.28)
PVF (0.278 mg/ml)	13.09(3.67)
PVF (0.019 mg/ml)	12.31(2.04)
Untreated	13.02(2.58)


SD; Standard deviation.

### Chromosome aberration test

In the case of the CA test, we observed insignificant differences in the MI between the PVF groups and negative control. On the other hand, both positive control agents (MMC and CP) demonstrated lower MI values compared to other groups. Those percentages of MI were significantly different when compared to the untreated (negative) control (reduction in more than 50% of MI, Table 2). No dose relationship was observed.

**Table 2:** Mitotic index (MI) of dental pulp stem cells (DPSCs) treated with perivitelline fluid (PVF)


Groups	Hours	Mean mitotic index (SD)(%)^a^		
		Without S9		With S9

PVF (26.887 mg/ml)	4	3.65(0.07)		3.60 (0.14)
	24	3.25(0.21)		-
PVF (14.093 mg/ml)	4	3.90(0.14)		3.55 (0.21)
	24	3.50(0.00)		-
PVF (0.278 mg/ml)	4	3.80(0.14)		4.20 (0.14)
	24	3.60(0.14)		-
PVF (0.019 mg/ml)	4	3.70(0.28)		3.60 (0.28)
	24	3.45(0.07)		-
MMC^b^	4	1.15(0.21)*		-
	24	1.25(0.21)*		-
CP^b^	4	-		1.70 (0.28)*
Negative control(culture medium)^c^	4	3.50(0.14)		4.40 (0.14)
	24	3.30	(0.28)	-


^a^; Mean from triplicate tests, ^b^; Positive controls mitomycin C (MMC) at a concentration of 0.1 μg/ml for 4 hours and 0.05 μg/ ml for 24 hours without S9 mix and cyclophosphamide monohydrate (CP) at a concentration of 10 μg/ml for 4 hours with S9 mix, ^c^; MSC basal medium, ^*^; P<0.05, MI is significantly different compared to the negative control and SD; Standard deviation.

**Fig.4:**
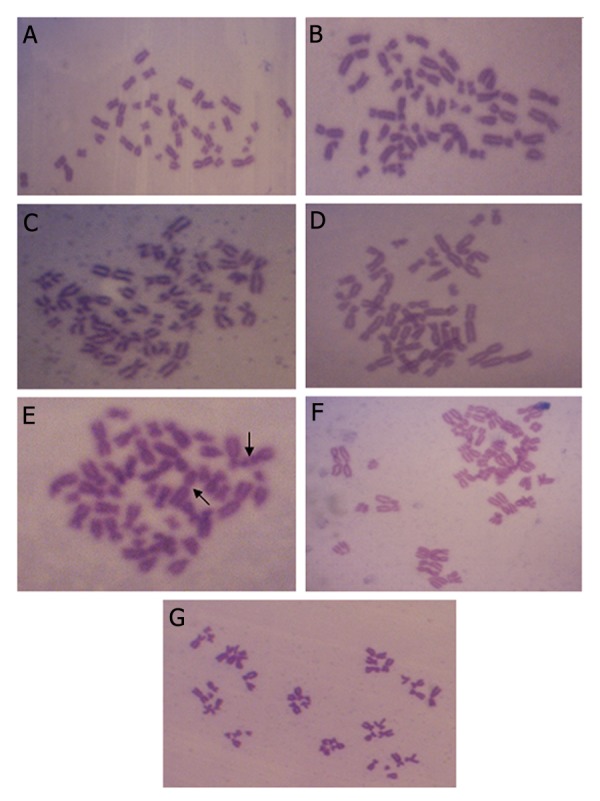
Representative images of cells treated with A. 26.887 mg/ml perivitelline fluid (PVF), B. 14.093 mg/ml PVF, C. 0.278 mg/ml PVF, D. 0.019 mg/ml PVF, E. Mitomycin C (MMC), F. cyclophosphamide monohydrate (CP) and G. Negative control. No significant chromosome aberrations (CA) were observed in PVF groups and negative control. Arrows show the gaps in the chromosome of cells treated with MMC. Formation of endoreduplication was seen in cells treated with CP.

Regardless of the treatment conditions (with and without S9 mix), different concentrations of PVF and duration of the treatment (4 and 24 hours), there was an absence of significant gross aberration in the chromosomes of the treated cell lines with PVF extract compared to the negative control. In contrast, multiple chromosomal abnormalities were seen in the both positive control groups (MMC and CP). The observed aberrations included chromosomal gaps, breaks, dicentrics, loss of centromeres and endoreduplications. Figure 4 shows the representative metaphase spreads from all groups.

### Ames test

In all triplicate tests, the results showed that the numbers of revertant colonies (TA98 and TA100 strains) treated with various PVF concentrations were less than 2-fold of the positive control in both treatment conditions (presence and absence of S9 mix, Table 3). The interpretations of these results were based on previously mentioned non-statistical analyses. No dose-response relationship was observed.

**Table 3:** Ames results of perivitelline fluid (PVF)


Groups	Average number of colonies^a^			
	TA98		TA100	
	With S9	Without S9	With S9	Without S9

PVF (14.093 mg/ml)	9 (2.08)	13(10.12)	22(9.61)	27(10.41)
PVF (7.045 mg/ml)	9 (3.51)	16(6.93)	32(8.54)	24(4.04)
PVF (3.523 mg/ml)	8 (1.15)	18(8.54)	23(5.03)	25(4.04)
PVF (1.761 mg/ml)	9 (2.89)	15(5.29)	21(4.73)	24(3.21)
PVF (0.881 mg/ml)	11 (3.61)	16(7.94)	26(3.51)	24(9.71)
Positive control^b^	37 (11.14)	65(11.37)	92(12.06)	175 (21.36)
Negative control (ddH_2_O)	10 (3.51)	12(4.04)	28(4.58)	27(3.46)


^a^; Average from triplicate tests, ^b^; Positive controls: 4-nitro-o-phenylenediamine (4-NoPD) and sodium azide (NAN3) for strains TA98 and TA100, respectively.

## Discussion

One of the reasons for the reduction in the population of the horseshoe crabs with regards to T. gigas in Singapore is the unavailability of sites that can support a breeding population (25). These arthropods have also been collected for extensive medical research (26). Massive interest in the species has stemmed from the discovery that its blood coagulates in the presence of minute quantities of gram-negative bacterial endotoxin (27). Over the last few decades, researchers have tried to explore the functions of other major components of horseshoe crabs, namely PVF. The protein components such as hemocyanins and lectins in PVF are proposed to be used in various biomedical areas such as immunology, embryology and tissue or cell engineering (28).

In the MTT assay, a greater cytotoxic effect caused by more concentrated PVF compared to diluted extract might be caused by the differences in the pH of the extract which might not be suitable for cell growth and proliferation. The possibility existed that various unidentified components in the crude extract of PVF might contribute to the production of inhibitory effects on cells treated with PVF.

The differences in the proliferation of DPSCs of treatment groups and the negative control seen in AlamarBlue® assay were attributed to the concentrations of PVF. This was supported by a study which deduced that for the enhancement of *in vitro* growth and activity of MSCs, the culture medium might be supplemented with proteins and factors in order to mimic the physiologic environment in which cells showed optimal proliferation and differentiation activity. Metabolism of cells in an organized environment was shown to be majorly associated with the intercellular metabolic interactions between different types of cells (29, 30). Regulation of stem cell behavior in natural and micro-engineered environments by exposure to selected mitogens and morphogens might also contribute (31, 32).

Rich nutrients in the PVF may affect cell proliferation. A previous study has stated that isolation of a 450 kDa multimeric lectin consists of a 40-kDa subunit from the PVF of *T. gigas* by using affinity chromatography of bovine submaxillary gland mucin-agarose (8). Ghaskadbi et al. (33) have reported that a constituent of PVF from the Indian horseshoe crab’s embryo can enhance growth and differentiation of a chick embryonic heart. Further purification of the factor involved in PVF of the horseshoe crab has led to identification of a cardiac promoting molecule, lectin. Proteins termed lectins refer to the partners that bind specific carbohydrate structures. Lectins are ubiquitous and can be found in animals, plants, and microorganisms. The main function of lectins in animals is to facilitate cell-cell contact. This interaction can be detected between the binding sites of lectins on the cell surface with arrays of carbohydrates located on the surface of the other cell (34). Many studies have demonstrated the effect of lectins on the proliferation of both normal and cancer cells (35-37). Lectins have also been discovered to produce proliferation activity on human peripheral blood mononuclear cells (38) and lymphocytes (39). The current study showed that upon treatment with PVF, proliferation of DPSCs increased on selected days which was probably due to the presence of lectins/proteins associated with the enhancement of cell proliferation.

As for the determination of PDT, even though there were slight reductions in the PDT of the PVF groups compared to the negative control, no significant differences were found in PDT in a comparison of both treatment and negative control groups. This indicated that the treated cells divided at almost the same rate as the untreated cells. It seemed that the components in the crude extract of PVF did not promote the cell division process and thus no significant decrease in PDT was produced. The differences in the PDTs of treatment groups were observed due to the effect of various PVF extracts. A concentrated PVF extract which produced the IC_50_ value produced a higher PDT compared to other PVF and negative control groups. This indicated a slower rate of cell division in the treated cells due to the high concentration of PVF. This effect might be attributed to the reduction in pH of the extract which did not serve as an optimum condition for cell proliferation compared to a more diluted PVF extract.

Through the CA test, no significant chromosomal aberrations were found in the PVF groups which showed the lack of genotoxic effect of PVF. This result differed from the positive control groups whereby various aberrations were observed. These results showed the genotoxicity effects of both MMC and CP. The significant reduction in the MI in positive control groups compared to the negative control also indicated the cytostatic effect of MMC and CP to the treated cells. The cytotoxicity and genotoxicity of both positive controls in the CA test were reported by other studies (40-43).Two treatment durations (4 and 24 hours) and different concentrations of positive controls were applied in the present study as reported previously by Hori et al. (44).

In the Ames test, even though the standard concentration values suggested by Mortelmans and Zeiger (24) ranged from 313 μg/ ml to 5000 μg/ ml, the concentration of 14.09 mg/ml PVF obtained from the initial cytotoxic study was used in order to determine the mutagenicity of the test material at that particular concentration of toxicity. This was further explained in the OECD guideline (22) which stated that testing for the concentrations of the material of more than 5 mg/plate or 5 ml/plate may be considered when evaluating substances that contained substantial amounts of potentially mutagenic impurities. OECD also stated that the requirement for at least five different analyzable concentrations of the test substance should be included in the Ames test (22) which justified the use of different doses of PVF extract.

Two different bacterial strains (TA98 and TA100) were selected for the present research as performed previously by Jurado et al. (45). The tester strains for Ames test are not isogenic and that genetic differences at loci other than his may be significant for mutagenicity testing. From Table 3, the results show no mutagenic activity in both bacterial strains even with the treatment of high concentration of PVF extract.

The identification of mutation-inducing agents is a crucial aspect in the safety assessment procedure. Chemicals that potentially cause mutations may result in fertility problems and the occurrence of mutations in future generations by damaging the germ line (24, 46). Mutagenic chemicals have the capability to induce cancer and this concern has been the focal point of most mutagenicity testing programs. Mutations can happen as gene (point) mutations where modification occurs at a single base, or insertion or deletion of one or a relatively few bases, large deletions or DNA rearrangements, chromosome breaks or rearrangements, or gain or loss of whole chromosomes (24).

The non-significant effect of this material on DPSCs in the current research could be attributed to the nature of the test material. In the present study, the crude extract was used for the test as opposed to the purified components such as proteins or peptides from PVF. To date, there has been limited information on the individual constituents of PVF from the horseshoe crabs. This served as a limitation for the study.

## Conclusion

Crude PVF from horseshoe crabs slightly increased viability of the cells. However, insignificant proliferative activity on DPSCs treated with PVF was produced. The absence of chromosomal aberrations in treated cells and negative result from Ames test indicated the non-genotoxic nature of PVF.
